# The Use of Microvolt T-Wave Alternans in Chagas Disease

**DOI:** 10.5935/abc.20180081

**Published:** 2018-05

**Authors:** Carlos Alberto Pastore

**Affiliations:** Instituto do Coração (InCor), Hospital das Clinicas FMUSP, Faculdade de Medicina, Universidade de São Paulo, São Paulo, SP - Brazil

**Keywords:** Chagas Disease, Electrocardiography, Cardiac Death, Sudden, Cardiac Risk Stratification, Cardiac Complexes, Premature

Visible T-wave *alternans* (TWA) has been reported since 1909, being, thus, not a recent finding.^1^ However, non-visible and far less rare microvolt TWA has gained importance because of its association with electrical disorders and the high risk for sudden cardiac death (SCD) or arrhythmic events,^2^^-^^6^ being assessed in several clinical trials and population studies, such as TWA in HF,^7^ ALPHA Study,^8^ REFINE Study,^9^ FINCAVAS,^10^ the collaborative study by Ikeda et al.^11^ and the MADIT-II-type research by Bloomfield et al.^12^ All those studies have in common the fact that they evidence the high negative predictive value of TWA regarding SCD or arrhythmic events, with low to intermediate positive predictive value.

A specialized software is used to analyze the microvolt TWA, the beat-to-beat variability that occurs in ventricular repolarization (ST segment and T wave) and that cannot be seen by the naked eye.^13^^-^^16^ The TWA allows indirect access to the increase in the dispersion to the action potentials of cardiac cells, a primordial factor in a sequence of events that will lead to reentry mechanisms and ventricular fibrillation, which will culminate with SCD. A fundamental property of its analysis is the high negative predictive power for the SCD risk that a normal TWA test has.^12^

Of the different methodologies to assess TWA, the two most used and relevant techniques in medical literature are: the spectral method (SM) and the modified moving average (MMA) method.^17^

The SM measures T-wave fluctuations by computing the point to point differences between 128 equally spaced sites in the ST-T complex, in a series of 128 consecutive aligned beats (having already ruled out ectopic beats and ECG noise).^18^ There are 128 tachograms similar to those used in the analysis of heart rate variability. Then, 128 heart rate variability spectra, hence the name of the methodology, SM, are computed, and their mean is calculated. The value of TWA is then assessed at the frequency of 0.5 cycle per beat. In 1994, the adaptation of that technique to human patients was published for the first time.^19^ Since then, SM is the most used method to analyze TWA, with the widest range of applications.

The MMA method repeatedly creates two patterns (models) of beats from any sequence of valid beats, one pattern associated only with the even beats, and the other associated with the odd beats. To clarify each pattern of the beats, the algorithm is as follows: the differences of amplitude between the current pattern (even or odd beats) and the next valid beat (even or odd) are measured along several equally spaced sites in the ST-T complex. Each of those differences is divided into X equal parts (where X can be 8, 16, 32 or 64), and the contribution of the current valid beat in the update of the standard beat is then limited to 1/X (named ‘the update factor’ or ‘limiting fraction’) of the differences between the model and the beat. Finally, the TWA values are made available every 15 seconds, as the difference between two representative patterns (and continuously updated) of the even beats and the odd beats.^20^ That technique has been assessed in academic studies with good reproducibility.^21^

In a study of Chagas disease, published in this issue of the *Arquivos Brasileiros de Cardiologia*,^22^ patients with chronic Chagasic cardiomyopathy and history of malignant ventricular arrhythmia most often had a non-negative result of microvolt TWA as compared to those with no previous arrhythmia, suggesting that TWA can play a role in the SCD risk stratification in Chagas disease. That study used the Cambridge Heart software with special electrodes (high resolution) and the SM. Its results are shown in terms of negative and non-negative (positive + indeterminate) TWA, the latter being compared to the former. That study emphasizes that Chagas cardiomyopathy has a true arrhythmogenic substrate confirmed by TWA.
